# Correspondence between F. J. S. Gorgas, Dean of University of Maryland Dental Department, and Dr. T. S. Waters, President of National Association of Dental Examiners

**Published:** 1888-10

**Authors:** 


### Editorial, Etc.

---

Correspondence between F. J. S. Gorgas, Dean of University of Maryland Dental Department, and Dr. T. S. Waters, President of National Association of Dental Examiners.—

Baltimore, October 9th, 1888.

*Dr. T. S. Waters, President National Association of Dental Examiners:*

*Dear Sir.*—On reading the proceedings of the Seventh Meeting of the "National Association of Dental Examiners," as published in the October number, 1888, of the *Dental Cosmos*, I was surprised to find that the "University of Maryland Dental Department" was not among the number of Dental Schools whose diplomas might be recommended by your Association to the various State Associations to be received in lieu of an examination. As the University of Maryland Dental Department has strictly complied with all the requirements of your Association in regard to the matriculation and graduation of Dental Students, length of sessions, &c., I desire to learn from you the cause of the omission.

Respectfully, &c.,

Ferdinand J. S. Gorgas,

Dean of University of Maryland Dental Department.

---

Baltimore, October 11th, 1888.

*Dr. F. J. S. Gorgas, Dean of University of Maryland Dental Department:*

*Dear Sir.*—In answer to your query of October 9th, as to the cause of the omission of the University of Maryland Dental Department from the list of Colleges and Departments to be recommended to the State Boards of Dental Examiners in lieu of an examination, have to say that it was by the unanimous action of the National Board of Dental Examiners. I do not call to memory the wording of the resolution, but if you will address Dr. Fred. A. Levy, of Orange, N. J., you can get the desired information. Dr. L. is the Sec. and has the records. Your School is not the only one left out in the cold. I think all Colleges that are not members of the Association of Dental Faculties were left out of the list. We also struck from the list one of the Washington Colleges that joined the Association of Faculties this year.

The National Board are honest in their work, and mean to maintain a high standard of Dental Education.

Very respectfully,

T. S. WATERS,

Pres. of N. B. of D. Examiners.

---

BALTIMORE, October 13th, 1888.

*Dr. T. S. Waters, Pres. Nat. Association of Dental Examiners :*

*Dear Sir.*—I desire to acknowledge the receipt of your reply to my communication of 9th inst. requesting of you the cause of the omission by your Association, of the name of the University of Maryland Dental Department from the list of Dental Schools whose diplomas have been recommended to the various State Associations to be received in lieu of an examination. In your reply you give as a reason for such omission, *that our Dental Department is not a member of the "Association of Dental Faculties,"* and also state that this action on the part of your Association was unanimous. Of course you mean that it was the unanimous action of the *seventeen members* who were alone present at the Louisville meeting of your Association on the 29th of August last, representing but *eleven* States—eight Northern and three Southern, including among the latter Maryland, from which you were the only member of the State Board of Dental Examiners present.

I desire to reply to your letter in the kindest manner possible, and without the slightest ill-feeling towards any one of the *seventeen* members, and I trust that you will so receive it, although I cannot help regarding such action as an indignity very unjustly and without cause placed upon one of the most reputable of the Dental Schools of this country, which has, from the date of its organization, maintained as high a standard of dental education as any other, and indeed a very much higher one than has many of the Schools whose diplomas the seventeen members of your Association recommend in lieu of an examination.

I think I am correct in stating, that (with one exception) I have been longer engaged in teaching dentistry than any other gentlemen now connected with the various dental schools of our country, and my aim has always been to maintain a high standard of Dental Education; hence, I can truthfully assert that I, as well as the gentlemen associated with me in the faculty of the University of Maryland Dental Department, have at all times been ready and willing to do all in our power to advance the interests of our profession, and aid those engaged in a like work.

Would the mere fact of membership in the Association of Dental Faculties give to our Dental Department any higher reputation than it at the present time possesses?

Has not our Dental Department from its organization, maintained as high a standard of dental instruction as any Dental School or Department in the world? and is not this fact acknowledged by every State Dental Association in this country who has recommended students to our School? And is it not further verified by the large number of dental students who attend its annual sessions, not one of whom has gone elsewhere to graduate, except those, who have either been rejected at the final examinations, or have been guaranteed diplomas, or lower fees, by other schools? Also by the large number of prominent and reputable dental practitioners of this and foreign countries who send their students to our Dental Department? Take the present unusually large class as an example, which contains members from England, Germany, Switzerland, Holland, Portugal, Turkey, South America, West Indies, Canada, New Brunswick, and from nearly every State of the United States. Again, have there ever been any charges brought against our

Dental Department for failure to comply with any of the requirements of the State Boards of Dental Examiners, or of the American and Southern Dental Associations? I have never heard of any, and defy anyone to prove that we have not strictly complied with all such requirements, and refer to our Annual Catalogues as to what our requiraments are. Have you or any member of the Maryland Board of Dental Examiners, with one exception, (Dr. Richard Grady), ever visited the Dental Department of the University of Maryland during its sessions, to make yourself acquainted with the manner of its teaching, although its doors have been, and ever will be open to you and your colleagues? Does not the fact that no less than *three* of the five members composing the Maryland Board of Dental Examiners, have recommended students to our Dental Department for the present session, and that Dr. Richard Grady, one of these members, has been sending students to us for several years, prove that the majority of the members of the Maryland Board of Dental Examiners are satisfied with our course of instruction and recommend it? Is not Dr. Richard Grady one of the most intelligent members of the Maryland Board of Dental Examiners, and fully competent to judge, having for many years filled the positions of Teacher and Principal in some of the highest Public Schools of Baltimore City? Have not the prominent dental practitioners from various parts of our country, who annually act as Judges in awarding prizes at the contests of our Graduating Classes, pronounced the operations performed in their presence by our Students in the Dental Infirmary, to be of the highest and most satisfactory character? Further: Is there any clause in the Maryland Dental Law that empowers you to act, in regard to said law, independent of your colleagues? For I have been informed that no meeting of your Board was held, at which you were appointed its representative to the Louisville meeting, and that no instructions were given you to omit the name of the "University of Maryland Dental Department" from the list of Schools whose diplomas were to be recommended. On the contrary, one of the members of your Board of Dental Examiners has repudiated such action, and declared it to be "*an outrage*," and "*a stab in the dark*." Was any intimation of such action on the part of the *seventeen* who met in Louisville, ever given to the Faculty of the Dental Department of the University of Maryland? I, its Dean, never heard of it until it appeared in the October number, 1888, of the *Dental Cosmos.* Does the Maryland Dental Law, or that of any State, empower its Board of Dental Examiners to discriminate against one of the Dental Schools in said State without any just cause? No court in our country will sustain such action; and you must know that the Maryland Board of Denlal Examiners cannot reject the diploma of the "University of Maryland Dental Department," merely because its Faculty have not become members of the Association of Dental Faculties; and the same may be said of every other State Board of Dental Examiners in this country.

ARTICLE VII of the Constitution of the "Association of Dental Faculties," reads as follows:

"Any reputable dental college may be represented in this body upon submitting to the Executive Committee satisfactory credentials, signing the Constitution, conforming to the rules and regulations of this body, and paying such assessments as may be made."

I am certain that you, regard our Dental Department as *a reputable* one, and the same may be said of every one of the members who composed the Louisville meeting, and who were a very small minority of the members composing the various State Dental Examining Boards.

In your opinion, is the restriction of dental students, while attending the dental schools of our country, to Mechanical Dentistry for the first year of the two-year's course, to the exclusion of Operative Dentistry for that year, a proper method of teaching dentistry? I am aware that this regulation has recently been made non-mandatory, but nevertheless the Association of Faculties yet recommend it. My long experience in teaching, leads me to consider a course of *two* year's instruction in operative dentistry as short enough.

Finally, I ask, if it was intended by the *seventeen* members composing the Louisville meeting, that printed circulars containing the names of the Schools whose diplomas are recommended to the various State Associations, and to which is attached the name of "Fred. A. Levy," Secretary, Orange, New Jersey, should be circulated among the students at present attending the "University of Maryland Dental Department," *as has been done*, for the purpose of injuring our Department? Several of these circulars addressed to our students, are now in my possession, but like all such mean acts, this one has failed in its purpose. I will do you justice by saying that I do not believe you have been a party to such an act, nor even aware of it; but that others have been thus guilty can be easily proven. We ask for our Dental Department no more than what is just, and are willing that it should be judged according to its merits; but we also demand that the members of the "Board of Dental Examiners," appointed by the Governor of the State of Maryland, shall act impartially, and foster, instead of injure, the reputable Dental Schools of Maryland.

Respectfully, &c.,

FERDINAND J. S. GORGAS,

Dean of University of Maryland Dental Department.

---

BALTIMORE, October 17th, 1888.

*Dr. T. S. Waters, Pres. Nat. Board of Dental Examiners:*

*Dear Sir* —I desire to acknowledge the receipt of your postal card of 16th inst., in which you state that "the National Board of Dental Examiners" took no action against the Dental Department of the University of Maryland, all they did being to recommend certain Schools as were or are members of the Association of Dental Faculties, and that "if your School had been a member it would be in the list recommended." I am very much gratified to learn this, especially for the reason that certain parties have taken advantage of this omission to circulate a report that our Dental Department is not recognized by the various State Boards of Dental Examiners, which, as you are aware, is false.

A letter, however, received this day from Dr. G. F. S. Wright, the President of the State Dental Examining Board of South Carolina, who was present at the Louisville Meeting of August 27th, 1888, where he represented his State Board, furnishes information concerning the proceedings of the National Association of Dental Examiners at the Louisville meeting, which is altogether different from the report published in the October number, 1888, of the *Dental Cosmos*, and also from the circular bearing the name of Fred. A. Levy, Sec'y., Orange, N. J., and which was mailed to Students of the Dental Department of the University of Maryland.

Dr. Wright's letter is as follows:

GEORGETOWN, S. C., October 15th, 1888.

*Professor F. J. S. Gorgas, Dean Dental Department University of Maryland:*

*Dear Sir.*—Your favor of the 9th has just reached me, it must have been delayed in Columbia. I do not know why the *Cosmos* is *short on facts* in the October issue, unless their reporter got his notes mixed. The said reporter was very busy with the regular joint meeting, as well as the separate meetings of the two Associations, and the Association of Faculties, also last but not least, The National Association of Examining Boards, *before which the Dental Department of the University of Maryland passed when the roll of Dental Schools was called. Passed without objection or comment of any kind.* If this had not been the case, and for cause it had been omitted, then I should have informed you, and if the cause could not be removed, then I should have requested a change in the Corps of Clinical Instructors. Dr. Waters, of your city was presiding when the above roll was called by the Secretary, and as each Institution was named the President would ask in these words: "*Any objection?*" and if none was offered, he announced: "*Passed.*', But if objection or inquiry arose, the merits of such case was then discussed and disposed of. If you ask Dr. Waters, of your city, who at that time was elected President of the National Association of Dental Examining Boards, or write to Dr. Fred Levy, of N. J., my statement will be sustained. If my memory is not at fault, there were but three Institutions objected to, one in Canada, and two in the United States, but not in Baltimore.

Very respectfully,

G. F. S. WRIGHT, D. D. S.

President of South Carolina State Board of Dental Examiners, and Delegate to the National Association of State Dental Examining Boards, Meeting, Louisville, Ky., 1888.

Respectfully, &c.,

FERDINAND J. S. GORGAS,

Dean of University of Maryland Dental Department.

BALTIMORE, October 19th, 1888.

*Dr. T. S. Waters, Pres. Nat Association of Dental Examiners:*

*Dear Sir.*—I desire to call your attention to the following letter received to-day from Dr. G. F. S. Wright, President of the South Carolina State Dental Examining Board:

GEORGETOWN, S. C., October 17th, 1888.

*Professor Gcrgas:*

Your telegram just received. If, after you have written to the Secretary of the National Association of State Dental Examining Boards, and received his answer, you find it necessary, you may publish the *fact*, that I say the Dental Department of the University of Maryland *was passed by the National Association with the same standing as the other Schools whose diplomas may be accepted as equivalent to an examination.*

Very truly yours,

G. F. S. WRIGHT, D. D. S.,

President of South Carolina State Board of Dental Examiners, and Delegate to Louisville, Ky., 1888.

Respectfully, &c..

FERDINAND J. S. GORGAS,

Dean of University of Maryland Dental Department.

---

BALTIMORE, October 29th, 1888.

*Dr. T. S. Waters, Pres. National Association of Dental Examiners:*

*Dear Sir:*—At your suggestion, and also that of Dr. G. F. S. Wright, President of the South Carolina State Board of Dental Examiners, I wrote to Dr. Fred. A. Levy, of Orange, N. J., asking him if our Dental Department had been omitted from the list of Dental Schools by your Association, and if so, for what cause? The only reply I received from Dr. Levy was that he understood I had printed a circular in which his name appeared, and requested a copy. He returned no answers whatever to my questions.

This day I received the following letter from Dr. W. C. Wardlaw, of Augusta, Georgia, a member of the Georgia State Board of Dental Examiners, who was also present at the

Louisville meeting of August, 1888, as a representative of his State Board:

AUGUSTA, GA., October 25th, 1888.

*Dr. F. J. S. Gorgas, Dean University of Maryland Dental Department:*

*Dear Doctor.*—I am just in receipt of a copy of the correspondence between yourself, Dr. Waters, and Dr. Wright, relative to the omission of the name of your College from the list of Colleges whose diplomas should be recommended to the Examining Boards in lieu of an examination.

I am glad that before its reception I had an opportunity of pronouncing, at a meeting of Dentists, the said omission a clerical error. I, with Dr. S. B. Barfield, of Macon, Ga., represented the Georgia Examining Board in the Louisville meeting, and my recollection is clear upon this matter. For having heard whisperings that objections might be made against your College, I, at considerable inconvenience, attended the Session of the National Board of Dental Examiners, at which the roll of Colleges was reviewed, prepared to fight the question, and was consequently upon the *qui vive.* The roll had been gone over at a previous meeting, and in being revised for adoption at this session, was gone through with two or three times, and each time there were errors to be corrected, so that I am not surprised if the mistake (unintentional) does actually exist in the Secretary's record. But a mistake it certainly is. I feel sure that Dr. Barfield and others will substantiate me in this.

There was one subsequent session of the Board of Dental Examiners at which I was not present, but at which, I was told, nothing had been done but the election of officers, and routine business.

I voluntarily write this, so that if the records of Secretary Levy are wrong, your College may still be set right.

Very truly yours,

W. C. WARDLAW.

Respectfully, &c.

FERDINAND J. S. GORGAS,

Dean of University of Maryland Dental Department.

BALTIMORE, November 1st, 1888.

*Dr. Fred. A. Levy, Sec'y. Nat. Asso. of Dental Examiners:*

*Dear Sir.*—I desire to acknowledge the receipt this day of a printed copy of the proceedings of the Seventh Annual Meeting of "The National Association of Dental Examiners," and, in return, send to you enclosed the printed circular, containing the correspondence between Dr. Waters, Dr Wright and myself, and also a letter from Dr. W. C. Wardlaw, of the Georgia State Board of Dental Examiners, who, with Dr. S. B. Barfield, represented said Board at the Louisville meeting of your Association. According to Dr. Wardlaw's letter, the list of Dental Schools was reviewed and re-reviewed two or three times, and finally disposed of, with no objections to the University of Maryland Dental Department, which, as Dr. Wright also states, was "*passed without objection or comment of any kind.*" In your report of the proceedings, according to the printed pamphlet received from you to-day, it appears that in the last session of your Association, at which there was a very much reduced attendance from the *seventeen* original representatives, but at which session the State Board of Georgia *was* represented, if that of South Carolina was not. The list of Dental Schools was again revised, and according to the number published in your printed proceedings the University of Maryland Dental Department was omitted. Is not such a record of your proceedings strangely incorrect, as Dr. G. F. S. Wright, a copy of whose letter I send you, was, according to your report, one of the Committee appointed to prepare a list of the Dental Colleges which your Association would recommend to the various State Boards? Does not Dr. W. C. Wardlaw state that although he was not present at the last session, yet he understood and was "told that nothing had been done but the election of officers and routine business." Yet your report alleges that the list of Colleges was again (notwithstanding the many previous reviews) "referred back to the Committee to be revised and was again presented to the Association; was amended and accepted."

The conclusions I am compelled to arrive at, from all the testimony given in the letters of Drs. Wright and Wardlaw, who declare that the University of Maryland Dental Department was not omitted from the list of Dental Colleges whose diplomas were to be recommended to the various State Boards (the former being a member of the Committee appointed to prepare said list of Dental Colleges), are—that you have either confounded the proceedings of the Second Session of your Association with those of the Third Session unintentionally ; or, that advantage was taken of the absence of several of the representatives who were present at the former sessions, to injure our Dental Department without any just cause.

Respectfully, &c.,

FERDINAND J. S. GORGAS,

Dean of University of Maryland Dental Department.

